# Hypertension of Developmental Origins: Consideration of Gut Microbiome in Animal Models

**DOI:** 10.3390/biomedicines10040875

**Published:** 2022-04-09

**Authors:** You-Lin Tain, Chien-Ning Hsu

**Affiliations:** 1 Department of Pediatrics, Kaohsiung Chang Gung Memorial Hospital and Chang Gung University College of Medicine, Kaohsiung 833, Taiwan; tainyl@cgmh.org.tw; 2 Institute for Translational Research in Biomedicine, Kaohsiung Chang Gung Memorial Hospital and Chang Gung University College of Medicine, Kaohsiung 833, Taiwan; 3 Department of Pharmacy, Kaohsiung Chang Gung Memorial Hospital, Kaohsiung 833, Taiwan; 4 School of Pharmacy, Kaohsiung Medical University, Kaohsiung 807, Taiwan

**Keywords:** developmental origins of health and disease (DOHaD), gut microbiota, hypertension, short chain fatty acid, oxidative stress, probiotics, prebiotics, renin–angiotensin system

## Abstract

Hypertension is the leading cause of global disease burden. Hypertension can arise from early life. Animal models are valuable for giving cogent evidence of a causal relationship between various environmental insults in early life and the hypertension of developmental origins in later life. These insults consist of maternal malnutrition, maternal medical conditions, medication use, and exposure to environmental chemicals/toxins. There is a burgeoning body of evidence on maternal insults can shift gut microbiota, resulting in adverse offspring outcomes later in life. Emerging evidence suggests that gut microbiota dysbiosis is involved in hypertension of developmental origins, while gut microbiota-targeted therapy, if applied early, is able to help prevent hypertension in later life. This review discusses the innovative use of animal models in addressing the mechanisms behind hypertension of developmental origins. We will also highlight the application of animal models to elucidate how the gut microbiota connects with other core mechanisms, and the potential of gut microbiota-targeted therapy as a novel preventive strategy to prevent hypertension of developmental origins. These animal models have certainly enhanced our understanding of hypertension of developmental origins, closing the knowledge gap between animal models and future clinical translation.

## 1. Introduction

Hypertension is the most common chronic disease and yields considerable morbidity and mortality globally [1]. Because of the multifactorial nature of hypertension, the use of various animal models, which evoke hypertension by different mechanisms, is advantageous for unraveling disease pathogenesis and developing novel antihypertensive drugs [2,3,4]. Though we are seeing tremendous progress on experimental hypertension, the prevalence of hypertension is still high and continues to rise worldwide [5].

Epidemiological and animal studies support that hypertension may be programmed in utero [6,7,8,9]. The association between fetal development and increased risk of adult disease has emerged as the concept of developmental origins of health and disease (DOHaD) [10]. A wide spectrum of early-life insults can evoke developmental programming resulting hypertension later in life. These insult stimuli include, but are not limited to, maternal malnutrition (both under- and overnutrition), maternal medical conditions, environmental exposure to toxins/chemicals, lifestyle changes, and medicines taken during pregnancy [7,8,9,11,12,13,14].

Over the past decade, the pathogenesis behind hypertension of developmental origins has not been fully elucidated, but data from animal models have proposed several key mechanisms [14]. Until now, the proposed mechanisms consist of aberrant renin–angiotensin system (RAS), oxidative stress, reduced nephron numbers, gut microbiota dysbiosis, dysregulated nutrient-sensing signals, sex differences, epigenetic regulation, etc. [7,8,9,11,12,13,14]. Among them, the interaction between the gut microbiota and the host implicated in hypertension has received significant interest [15,16,17,18]. Despite gut microbiota dysbiosis being observed in multiple animal models of hypertension [15,16], too little attention has been paid to its role in hypertension of developmental origins.

Although blood pressure (BP) is considered with a multifactorial pattern of inheritance, genome-wide association studies cumulatively could only explain ~3.5% of BP trait variability [19]. Accordingly, it is likely that the influence of environmental and epigenetic factors on the developmental programming of hypertension should receive wider recognition. Notably, maternal insults can impair gut microbiota composition and function, leading to adverse offspring outcomes later in life [20]. Conversely, review elsewhere indicted that early-life gut microbiota-targeted therapies have benefits on the prevention of the developmental programming of adult disease, including hypertension [21]. All this raises the notion that we need to pay more attention to prevent and not just treat hypertension, with a focus on the influence of dysbiotic gut microbiota on hypertension of developmental origins. Accordingly, animal models would likely be very useful in unraveling these actions.

In this review, we describe the role of gut microbiota implicated in animal models used for studying the developmental programming of hypertension. Therefore, we summarize the contributions of animal models linking the gut microbiota to developmental programming of hypertension, which helps in developing valuable strategies to prevent hypertension from happening. We specifically focus on addressing gut microbiota-targeted therapies such as probiotics, prebiotics, and postbiotics as a reprogramming strategy for prevention of hypertension of developmental origins.

In view of the above, a search was performed in the electronic bibliographic database PubMed/MEDLINE. Search terms were as follows: “developmental programming”, “DOHaD”, “animal model”, “pregnancy”, “gestation”, “offspring”, “progeny”, “prenatal”, “perinatal”, “mother”, “maternal”, “reprogramming”, “gut microbiota”, “probiotics”, “prebiotics”, “postbiotics”, “synbiotics”, “blood pressure”, and “hypertension.” Relevant abstracts were identified and reviewed to identify appropriate studies. Suitable published articles in English were included, without restriction of the time of publication.

## 2. Hypertension of Developmental Origins: Choice of Animal Models

**Figure 1:**
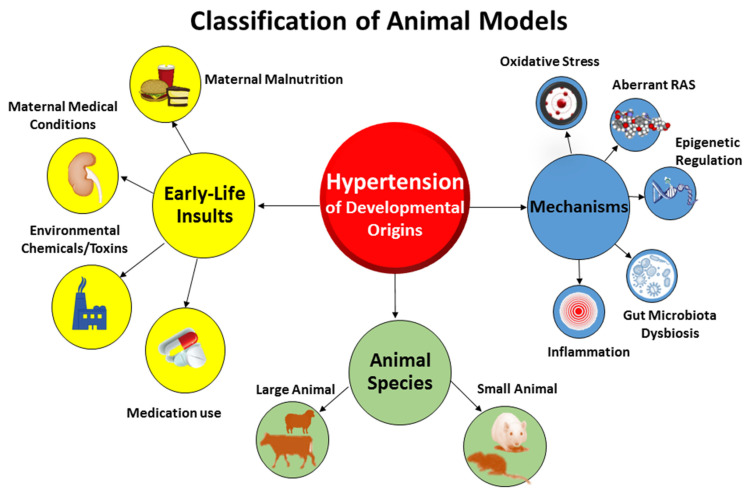
The classification of major animal models for studying hypertension of developmental origins.

Compared to animal models of essential hypertension established in prior research [2,3], the etiologies of hypertension of developmental origins and underlying pathogenic mechanisms are more complex [14]. Animal models of hypertension of developmental origins can be categorized in different ways (Figure 1).

Firstly, these models can be classified according to early-life adverse conditions. Nutritional programming is the most common type of animal model being studied in the field of DOHaD research [22]. Dietary caloric or protein restriction in animals mimics the starvation linked to famine in human cohorts [23,24]. Imbalance of maternal nutrition can have long-term changes in BP, resulting hypertension in adult offspring [25]. Inadequate or excessive intake of a specific nutrient has been established to induce hypertension of developmental origins in animal models, as reviewed elsewhere [11,26]. These models of undernutrition related to hypertension of developmental origins include, but are not limited to, caloric restriction [27], protein restriction [28], and deficiencies in sodium [29], calcium [30], zinc [31], iron [32], methyl donor nutrients (choline; vitamins B2, B6, and B12; folic acid; and methionine) [33], and vitamin D [34]. On the other hand, overnutrition characterized by the consumption of a high-fat [35,36], high-fructose [37,38], or high-protein diet [39] by rodent mothers also leads to early programming of hypertension in the offspring. Additionally, animal models resembling maternal medical conditions have also been evaluated in developmental programming of hypertension. These models include hypertensive disorders of pregnancy [40], preeclampsia [41], diabetes [42], chronic kidney disease (CKD) [43], maternal hypoxia [44], etc. Furthermore, chemical and medication exposures during pregnancy increase the risk of developing hypertension in offspring [13,14]. Prenatal exposure to 2,3,7,8-tetrachlorodibenzo-p-dioxin (TCDD) [45], bisphenol A [46], nicotine [47], caffeine [48], cyclosporine [49], gentamicin [50], tenofovir [51], minocycline [52], or glucocorticoids [53] has been reported to induce hypertension of developmental origins in various animal models.

Secondly, animal models can be classified based on molecular mechanisms. In view of different early-life adverse environmental factors producing the same outcome, that is to say hypertension in adult offspring, there might be core mechanisms underlying hypertension of developmental origins. These mechanisms include gut microbiota dysbiosis [21], oxidative stress [12], aberrant RAS [54], reduced nephron numbers [7], dysregulated nutrient-sensing signals [55], sex differences [56,57], epigenetic regulation [58], inflammation [9,14], nitric oxide (NO) deficiency [59], etc. Up to date, various animal models have been developed to test such proposed mechanisms. Because of the multifactorial nature of developmental hypertension, the use of various animal models, each of which induces hypertension by a different mechanism yet with the same end result, is advantageous. This approach would allow for a novel and effective reprogramming intervention targeting a specific molecular pathway to be adopted for preventions and therapies.

Lastly, animal models in DOHaD research can be classified according to species [60]. Diverse large- and small-animal models have been used, each with its own natural advantages and disadvantages [8]. Former reviews demonstrated that cow [61], sheep [62], rat [27], and mice [63] have be used to study hypertension of developmental origins [14]. Considering that rat models are cost-effective and easy to maintain and breed, they became the most common species used in the research field of DOHaD-related hypertension [14]. Although nonhuman primates [64], swine [65], rabbits [66], and guinea pig [67] have been studied for cardiovascular outcomes induced by maternal insult stimuli, none of them have been reported for examining hypertension of developmental origins.

Rats are by far the most often used species in the field of primary hypertension research. Of these, the spontaneously hypertensive rat (SHR) without any doubt is the most popular strain [2]. However, the majority of the rat strains used for studying developmental hypertension are Sprague-Dawley (SD) or Wistar [14]. In view of the genetic background of SHR, offspring develops hypertension spontaneously without programming induced by early-life insults, weakening its application in studying hypertension of developmental origins. Hence, the choices of animal models between essential hypertension and hypertension of developmental origins are quite different. Many more aspects of animal models need to be taken into further consideration, such as the timing of organogenesis [68], life cycle [69], gestation period [70], litter size [71], offspring outcomes, than in human studies [72], and valuable therapeutic interventions need to be evaluated and validated [14].

Together, it is noted that remarkable advances in hypertension of developmental origins have been originated from animal models. However, what is missing in the literature is animal models used for studying hypertension-related complications. Although elevated BP is the core feature of human hypertension, its morbidity and mortality occur with complications in the heart, brain, kidneys, and vessels. The contributions of early-life insults to these hypertension-related complications later in life in an organ-dependent manner have not yet been well-studied in the above-mentioned animal models.

## 3. Gut Microbiota: Choice of Animal Models

Trillions of bacteria living in the gut—the gut microbiota—coexist with the host in a mutually beneficial relationship [73]. Microbiota refers to all the microorganisms found in the environment, while the term microbiome refers to the collection of genomes from all microorganisms in a given environment. A variety of environmental factors can cause the disturbance of gut microbiota (i.e., dysbiosis), which in turn can influence human health and disease. Although the influence of gut microbiota in hypertension has been extensively reviewed elsewhere [15,16,17,18], less attention was paid to exploring its role in hypertension of developmental origins.

Directly after birth, microbes colonize the neonatal gut immediately [74]. These alterations continue until three years of age and mediate the transition toward an adult-like gut microbiota [75]. During pregnancy and lactation, the mothers share gut microbes and microbial metabolites with their offspring, which highlights the importance of maternal influences in the development of early-life gut microbiota [76]. A diversity of early-life factors governs the establishment of the gut microbiota, such as maternal medical conditions, gestational age, types of delivery, antibiotic exposure, formula feeding, and ecological factors [74,75,76,77].

**Figure 2:**
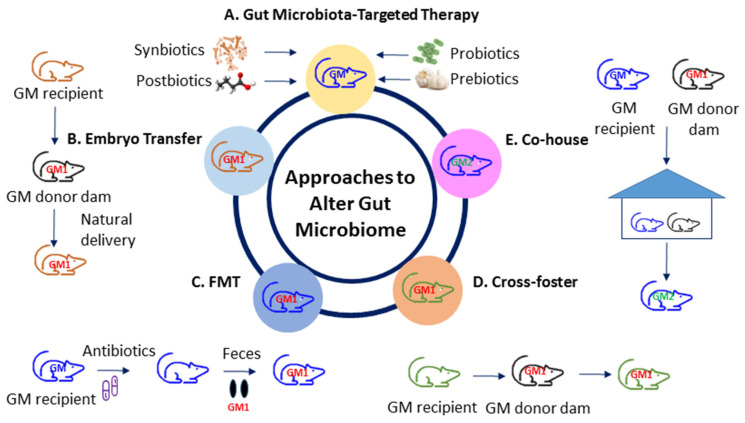
Different approaches to altering the gut microbiota. (**A**) Gut microbiota-target therapy; (**B**) embryo transfer; (**C**) fecal microbiota transfer; (**D**) cross-foster; (**E**) co-house. FMT = fecal microbiota transfer; GM = gut microbiota; GM1 = transferred gut microbiota; GM2 = GM + GM1.

So far, animal models have been broadly established to investigate human diseases in gut microbiota research [78]. Figure 2 illustrates various approaches to alter the gut microbiota in animal models of disease. Several gut microbiota-targeted therapies have been used to alter gut microbiota compositions and its derived metabolites. These interventions consist of probiotics, prebiotics, synbiotics, postbiotics, etc. [14]. The embryo transfer (ET) method is considered the gold standard for gut microbiota transfer. Additionally, researchers often use other methods to transfer the gut microbiota, such as fecal microbiota transfer (FMT), co-housing (CH), or cross-fostering (CF) [78].

Several gut microbiota-targeted therapies have shown to alter the gut microbiome. Probiotics (i.e., live beneficial microbes) and prebiotics (i.e., substances in foods that promote the growth of healthy microbes) are the most commonly used gut microbiota-targeted modalities in clinical practice [79]. Synbiotics refer to a mixture comprising probiotics and prebiotics that also confers a health benefit [79]. In addition, the use of substances leased or produced through gut microbial metabolism, namely postbiotics, have shown an influence on gut microbiota compositions and metabolites [80].

In the approach to transfer embryos, they are collected from the gut microbiome recipient and surgically transferred to a pseudopregnant donor dam [78]. Accordingly, the recipient pups obtain the vaginal microbiota from the donor dam through vaginal delivery. Nevertheless, this method needs considerable costs and expertise, making it inaccessible for many labs. Using the FMT approach, feces or fecal contents from donors are transferred to recipient animals via gastric gavage. Germ-free mice or antibiotics-treated depleted microbiota animals are commonly used as recipients [81].

Another commonly used method is CH, wherein recipients are co-housed with a donor after weaning [82], leading to the transfer of the donor gut microbiome through coprophagy and grooming [82]. Although the co-housing approach is easy and low-cost, the transfer of the gut microbiota after the critical developmental period results an incomplete transfer as well as a hybridized gut microbiome. When the recipient pups are housed in cages with the donor dam within 24 h after birth, the CF method allows the recipients to obtain most of their gut microbiota from the donor dam [78]. Compared to CH, the CF approach transfers the gut microbiota from an early age during the maternal care process.

All these methods each carry certain advantages and limitations. Researchers should thus be mindful of these method-related differences in the context of the transfer methods used for studying the role of the gut microbiota on hypertension of developmental origins.

## 4. Gut Microbiota in Hypertension of Developmental Origins

There is mounting evidence to support the pathogenic interconnection between the gut microbiome and hypertension [15,16,17,18]. However, there is paucity of information regarding the influence of the gut microbiota on the developmental programming of hypertension later in life. Therefore, most data obtained from patients with established hypertension and knowledge received from animal models of essential hypertension might be extrapolated to hypertension of developmental origins.

### 4.1. Gut Microbiota and BP Regulation

A great deal of work on the influence of the gut microbiota and its derived metabolites on BP regulation has been conducted. First, data from several genetic hypertensive rat models (e.g., SHR) indicated that the gut microbiota of hypertensive rats is dysbiotic and significantly different from the microbiota of normotensive control rats [15]. Gut microbiota dysbiosis was also noted for other hypertension models such as animals treated with high salt [83], angiotensin II [84], and deoxycorticosterone acetate-salt [85]. Another line of evidence comes from germ-free animals. The absence of microbiota in germ-free rats resulted with relative hypotension compared with their conventionalized counterparts, suggesting an essential role of gut microbiota in BP regulation [86]. Additionally, germ-free mice that received FMT from a hypertensive human donor developed a gut microbiota similar to that of their donor, as well as elevated BP [87]. There are observations that microbial metabolites are involved in BP homeostasis. Short chain fatty acids (SCFAs) are the main metabolites produced during bacterial fermentation of carbohydrates. SCFAs are generally known to regulate BP via activating their SCFA receptor, including olfactory receptor 78 (Olfr78), G protein-coupled receptors (GPR) GPR41, GPR43, and GRP109A [88]. Another example is trimethylamine-N-oxide (TMAO). TMAO is a small colorless amine oxide produced by gut microbiota metabolism [89]. A high TMAO level correlates with CVD mortality [90]. Fourth, the uses of probiotics [91] or prebiotics [92] have shown benefits on hypertensive patients.

### 4.2. Animal Models Linking Gut Microbiota Dysbiosis to Hypertension of Developmental Origins

Much work investigating the actions of the gut microbiome has directly studied the hypertension models, yet relatively little data exists on its programming effect related to hypertension of developmental origins. A summary of animal studies indicating the association between dysbiotic gut microbiota and developmental hypertension in adult offspring is provided in Table 1 [40,52,93,94,95,96,97,98,99,100,101,102,103,104,105,106].

The current review is only restricted to early-life insults starting in the pregnancy and/or lactation period. Table 1 shows that rats are the most common species being used. A variety of early-life insults have been reported to induce developmental hypertension, accompanying alterations of the gut microbiota, including a maternal high-fructose diet [93,94,95], maternal high-fructose diet plus TCDD exposure [96], maternal high-fat/high-cholesterol diet [97], maternal high-fat and/or post-weaning high-fat diet [98,99], gestational hypertension [40,100], maternal CKD [43], maternal dyslipidemia [101], maternal N^G^-nitro-L-arginine-methyl ester (L-NAME) administration plus postnatal high-fat diet [102], maternal administration of minocycline [52], maternal TMAO and asymmetric dimethylarginine (ADMA) exposure [103], maternal TCDD exposure [104,105], and prenatal androgen exposure [106].

Table 1 lists the timing of hypertension determined from rat models, with age ranging from 12 weeks to four months. As every month of an adult rat corresponds to three human years [55], the observed periods correspond with humans from childhood to early adulthood.

### 4.3. Gut Microbiota Dysbiosis in Hypertension of Developmental Origins

**Figure 3:**
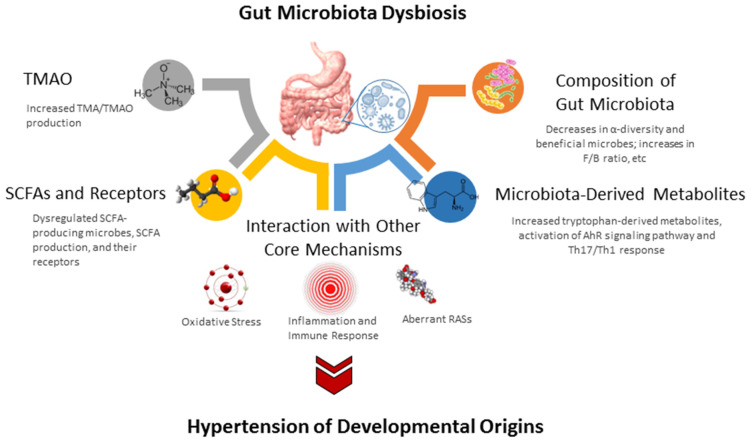
Overview of the gut microbiota and potential molecular mechanisms related to hypertension of developmental origins. SCFA short chain fatty acid. TMAO = trimethylamine N-oxide; TMA = trimethylamine; SCFA = short chain fatty acid; RAS = renin-angiotensin system; TH17 = T helper 17 cells; TH1 = T helper 1 cells; F/B ratio = *Firmicutes* to *Bacteroidetes* ratio; AhR = aryl hydrocarbon receptor.

The study of the gut microbiome in animal models of developmental hypertension mainly focuses on four types of dysbiosis: loss of diversity, decreases in beneficial microbes, shifts in key taxa, and alterations of microbial metabolites. A schematic summarizing the gut microbiota and a possible molecular pathway linked to hypertension of developmental origins is presented in Figure 3.

#### 4.3.1. Alterations in Gut Microbiota Compositions

First, α-diversity is decreased in models of maternal high-fat and high-cholesterol diet [97] and maternal TCDD exposure [104,105]. A similar pattern of gut dysbiosis was reported in several hypertensive animal models [15]. Second, a maternal plus post-weaning high-fat diet programming offspring’s hypertension coincides with an increased *Firmicutes* to *Bacteroidetes* (F/B) ratio and a reduction of genera *Lactobacillus* and *Akkermansia* [98,99]. This was found to be consistent with hypertension models showing the F/B ratio was increased and served as a microbial marker of hypertension [15]. Likewise, the increase of the F/B ratio is noted in other models of developmental hypertension programmed by a variety of maternal insults, including CKD [43], minocycline administration [52], hypertension [100], L-NAME administration plus high-fat diet [102], and TCDD exposure [104,105]. Both *Akkermansia* and *Lactobacillus* are known as one of the beneficial probiotic bacterial strains [107,108]. Decreases of certain beneficial microbes were also found in developmental models of hypertension, like maternal minocycline administration [52], maternal high-fructose diet [94], maternal hypertension [40], maternal dyslipidemia [101], and maternal TCDD exposure [104,105].

#### 4.3.2. SCFAs and Their Receptors

Notably, an association between microbiota-derived metabolites and hypertension has been found in several models of developmental hypertension [43,93,94,106].

SCFAs, the main metabolites produced by the gut microbiota, have one to six carbon atoms (C1–C6), mainly consisting of acetic acid (C2), propionic acid (C3), and butyric acid (C4) [88]. In SHR, hypertension is associated with decreased abundance of acetate- and butyrate-producing bacteria [15]. Similarly, SCFAs and their receptors are involved in hypertension of developmental origins, as reported in several animal models [52,93,106].

In a model of maternal administration of minocycline, minocycline-induced hypertension is associated with a reduction of plasma acetate and butyrate [52]. Another report demonstrated that dam rats receiving a 60% fructose diet caused offspring’s hypertension, coinciding with an increased plasma acetate level and a reduction of renal GPR41 and GPR43 expression [93]. As acetate is a ligand for GPR41 to induce vasodilatation, and Olfr78 exhibits vasoconstrictive action [109], these findings support the notion that SCFAs and their receptors contribute to maternal high-fructose-diet-induced hypertension in adult offspring. Additionally, maternal garlic oil therapy protects against offspring hypertension programmed by a high-fat diet, which is related to increased acetate, butyrate, and propionate, as well as their producing microorganisms [110]. Moreover, maternal SCFA supplementation have been reported to be protective on hypertension of developmental origins [85,94]. These findings support the notion that SCFAs and their receptors might be a crucial mechanism behind developmental programming of hypertension.

#### 4.3.3. TMAO

TMAO is an end-product of microbial carnitine and choline metabolism [89]. TMAO is converted from trimethylamine (TMA) by flavin-containing monooxygenase (FMO). TMAO is able to activate nuclear factor-κB (NF-κB) signaling, enhance leukocyte-endothelial cell adhesion, and induce inflammatory gene expression, all of which are related to the development of hypertension [111].

Maternal exposure to TMAO results in hypertension in adult male offspring [103]. Conversely, microbe-dependent TMA and TMAO formation can be inhibited by 3,3-dimethyl-1-butanol (DMB), a structural analogue of choline [112].

In a maternal high-fructose diet model, maternal DMB therapy showed protection against hypertension in adult rat offspring, which was relevant to the reduction of TMA and TMAO levels [94]. Another study demonstrated that perinatal resveratrol therapy protected adult rat offspring against maternal CKD-induced hypertension, which was associated with a decrease of the TMAO-to-TMA ratio [113]. These observations suggest a pathogenic link between the TMAO metabolic pathway and hypertension of developmental origins.

### 4.4. Core Mechanisms Linking to Gut Microbiota

Considering that various early-life insults during fetal development produce the same outcome―hypertension in adulthood—there might be some core mechanisms involved in the pathogenesis of hypertension of developmental origins. A number of mechanisms so far have been proposed, such as aberrant RAS, oxidative stress, reduced nephron numbers, dysregulated nutrient-sensing signals, inflammation, sex differences, epigenetic regulation [7,8,9,11,12,13,14]. Among them, some are interconnected to gut microbiota dysbiosis and will be discussed in turn.

#### 4.4.1. Oxidative Stress

During pregnancy, the presence of excessive reactive oxygen species (ROS) under suboptimal in utero conditions may prevail over the defensive antioxidant system and compromise fetal development, leading to oxidative stress damage [114]. A review elsewhere indicated that there are various types of in utero insult stimuli linked to oxidative stress in mediating hypertension of developmental origins [115]. The main mechanisms underlying the actions of oxidative stress-related hypertension of developmental origins include increased ROS-producing enzyme expression [116], increased ROS formation [117], decreased antioxidant capacity [118], impaired NO signaling pathway [27], increased lipid peroxidation [119], increased oxidative DNA damage [43], and increased peroxynitrite production [120].

Data from several animal models listed in Table 1 shows that the connections between gut microbiota dysbiosis and oxidative stress may be involved in the pathogenesis of programmed hypertension, including maternal CKD [43], high-fructose diet [95], and high-fat diet models [110]. Gut microbial communities are able to elicit redox signaling and maintain host–microbiota homeostasis [121]. An imbalance in the redox state can lead to inflammatory responses and gut damage, resulting in gut microbiota dysbiosis. In a maternal CKD model, offspring developed hypertension related to increased oxidative stress and impaired NO signaling [43]. In a subsequent study, perinatal resveratrol therapy protected adult offspring against hypertension programmed by maternal CKD, accompanied with reshaping the gut microbiota and reducing oxidative stress concurrently [113].

Together, oxidative stress may work together with the gut microbiota under hypertension of developmental origins. More attention needs to be paid to evaluate how the gut microbiota interconnects with oxidative stress to elicit organ-specific programming processes behind hypertension, and whether antioxidant therapy in pregnancy may also benefit the gut microbiota to protect adult offspring against hypertension of developmental origins.

#### 4.4.2. Aberrant RAS

The RAS is a major regulatory network that maintains BP, and blockade of the RAS has emerged as a therapeutic option for hypertension [122]. An increasing number of animal models linked to aberrant RAS are now being developed to evaluate hypertension of developmental programming, as reviewed elsewhere [54].

Within the RAS, regulation is achieved through a cascade of proteases generating some bioactive peptides [122]. The classical RAS consists of angiotensin-converting enzyme (ACE), angiotensin (ANG) II, and angiotensin II type 1 receptor (AT1R). Activation of the classical RAS elicits vasoconstriction and inflammation under pathophysiological conditions, consequently resulting in hypertension and its related complications [123].

On the other hand, the nonclassical RAS is composed of ACE2/angiotensin-(1-7) (Ang-(1-7))/Mas receptor/ANG II type 2 receptor (AT2R), by which it can counterbalance the adverse effects of ANG II [124]. A growing body of evidence supports that aberrant RAS plays a key role in developmental hypertension, and RAS-based interventions can be used as a reprogramming strategy to prevent hypertension [54].

Mounting evidence suggests a bidirectional interaction between the gut microbiota and RAS; alterations in RAS shift microbiota composition and metabolic activity, while gut microbiota-derived metabolites can modulate the gut RAS [125]. Through regulation of intestinal amino acid transport, prior research reported that ACE2 plays a key non-catalytic role in gut biology and modulation of the gut microbiota [126].

Adult rat progeny of CKD mothers developed hypertension, coinciding with decreased expression of Mas receptor and AT2R [43]. In another high-fructose diet plus TCDD exposure model, 3,3-dimethyl-1-butanol (DMB) therapy protected against hypertension, coinciding with a reduction of AT1R but an increase of AT2R protein abundance, as well as reshaping the gut microbiota [96]. Other developmental hypertension models such as a perinatal high-fat diet [99], maternal administration of minocycline [52], and maternal TMAO plus ADMA exposure [103] also interfere with aberrant RAS and gut microbiota dysbiosis.

As gut microbiota dysbiosis has been linked to hypertension by modulating the systemic and local RAS [127], these observations endorse the idea that the interaction between the RAS and the gut microbiota implicates the pathogenesis of developmental programming of hypertension, although this remains speculative.

#### 4.4.3. Inflammation and Immune Response

Pregnancy is considered a physiologic systemic inflammatory response; compromised pregnancies and associated complications may be attributed to inflammation [128]. The accumulation of T cells, monocyte/macrophages, and T cell-derived cytokines is involved in the pathogenesis of hypertension [129]. An imbalance of T regulatory cells (Treg) and T helper 17 (TH17) cells has been linked to hypertension [129], which can be restored by postbiotic therapy [130]. In CKD, the interplay between Treg/TH17 balance and inflammation has also been related to hypertension [131]. Treg and TH17 cells can both be regulated by aryl hydrocarbon receptor (AhR) [132].

It is noted that several microbial tryptophan metabolites are uremic toxins as well as AhR ligands. AhR signaling can initiate inflammation through increasing monocyte adhesion, upregulating proinflammatory gene expression, reducing NO bioavailability, and inducing the expression of endothelial adhesion molecules [133]. Several gut microbiota-derived uremic toxins, like indoleacetic acid and indoxyl sulfate, have pro-oxidant, proinflammatory, procoagulant, and proapoptotic effects, all of which are involved in the pathogenesis of hypertension [134].

Using a rat model of maternal CKD-induced hypertension, we observed that maternal tryptophan therapy preventing offspring’s hypertension coincides with restoration of the AhR signaling pathway and several tryptophan-metabolizing microbes [134]. Another study showed that TCDD-induced programming hypertension is related to TH17-induced renal inflammation, the activation of AhR signaling, and alterations of gut microbiota compositions [105]. In contrast, TCDD-induced activation of AhR signaling and TH17 responses can be restored by perinatal supplementation with resveratrol, an AhR antagonist. Additionally, resveratrol is reported to have benefits on offspring hypertension in several developmental hypertension models [45,46,95,102].

Although results from animal models support the role of inflammation and immunity on hypertension of developmental origins, more research is required to gain comprehensive insight into their interconnections with the gut microbiota and develop therapeutic potential of inflammation- or immune-targeted therapies in hypertension of developmental origins and associated organ damage.

## 5. Reprogramming Strategy: Gut Microbiota-Targeted Therapy

The idea from DOHaD research creates opportunities to reverse the programming process, namely reprogramming, by early intervention aiming to prevent hypertension of developmental origins later in life [135]. Current literature on animal studies for hypertension of developmental origins supports that gut microbiota-targeted therapy can work as a reprogramming strategy to prevent hypertension induced by various early-life insult stimuli.

### Animal Models Used for Reprogramming

Here, we show Table 2 that summarizes studies documenting microbiota-targeted reprogramming therapies in animal models of developmental hypertension, restricting those starting before or upon disease onset [93,94,96,99,103,105,110,113,134,136,137]. The therapeutic duration is from pregnancy through lactation, which cover the periods of organogenesis. The literature review states that gut microbiota-targeted strategies used to prevent hypertension include probiotics, prebiotics, postbiotics, and dietary nutrients.

Table 2 illustrates that the most commonly used species are rats. Several models of developmental hypertension have been used to examine gut microbiota-targeted interventions, such as maternal high-fructose diet [93,94], perinatal high-fat diet [99,110,136], perinatal TCDD exposure [105], maternal adenine-induced CKD [113,134,137], maternal TMAO and ADMA exposure [103], and maternal high-fructose intake plus TCDD exposure [96].

Despite probiotics and prebiotics showing benefits in hypertension [91,92], there was very limited evidence in regard to their role on hypertension of developmental origins. Probiotic treatment with *Lactobacillus casei* in pregnancy and lactation prevents the development of hypertension in adult male rat offspring raised on a maternal high-fructose diet [93] or perinatal high-fat diet model [99].

As a prebiotic, inulin has a protective effect in hypertension of developmental origins [93,99]. A previous study using a maternal high-fructose model demonstrated that perinatal inulin treatment protects against offspring’s hypertension, accompanied by increased abundance of *Lactobacillus*, the most-known probiotic strain [93]. Another study demonstrated that perinatal supplementing with inulin protects against maternal high-fructose-diet-induced hypertension, accompanied by increases of plasma propionate concentrations [99].

Additionally, resveratrol could be used to protect against adult disease of developmental origins due to its probiotic properties [138]. Studies using a maternal TMAO plus ADMA exposure rat model indicate that adult rat progeny born to dams exposed to uremic toxins develop hypertension [103]. Nonetheless, maternal resveratrol therapy rescues from hypertension programmed by TMAO plus ADMA exposure, accompanied by increased butyrate-producing bacteria and fecal butyrate level.

Another study demonstrated that adult rat progeny born to dams exposed to TCDD have hypertension [105], which is related to activation of AhR signaling, induction of TH17-dependent renal inflammation, and shifts of gut microbiota compositions [105]. Conversely, the induction of TH17- and AhR-mediated inflammation can be counterbalanced by perinatal resveratrol supplementation. The beneficial effects of resveratrol are also relevant to reshaping the gut microbiota by augmenting microbes that can inhibit TH17 responses and reducing the F/B ratio. In a maternal CKD model, adult rat progeny developed renal hypertrophy and hypertension [82]. Perinatal resveratrol therapy protects from hypertension, accompanied by restoration of microbial richness and diversity and an increase in beneficial microbes, *Lactobacillus* and *Bifidobacterium* [113]. Nevertheless, the low bioavailability of resveratrol limits its clinical translation [139]. In this regard, we synthesized resveratrol butyrate ester (RBE) from resveratrol and butyrate by esterification to improve the efficacy [140]. We recently found that low-dose RBE (25 mg/L) is as effective as resveratrol (50 mg/L) in protecting against CKD-induced hypertension [141], despite the beneficial effect of RBE in models of developmental hypertension awaiting further evaluation.

Although there are many other prebiotic foods, only garlic oil has shown benefits on protection of perinatal high-fat-diet-induced hypertension in adult progeny [110]. The beneficial effects of garlic oil include increased α-diversity; increased plasma acetate, butyrate, and propionate; and increased beneficial bacteria *Lactobacillus* and *Bifidobacterium*.

In addition to probiotics and prebiotics, postbiotics are another gut microbiota-targeted modality. Postbiotics include various components like microbial cell fractions, extracellular vesicles, cell lysates, extracellular polysaccharides, functional proteins, cell wall-derived muropeptides, etc. [80]. Nevertheless, very limited information exists regarding the use of postbiotics in hypertension. As a postbiotic, acetate for perinatal supplementing showed benefits on maternal high-fructose-diet-induced hypertension [94].

Additionally, two studies demonstrated that maternal DMB therapy, an inhibitor of TMA formation, protects from hypertension in adult rat progeny exposed to maternal high-fructose diet with or without TCDD exposure [94,96]. Its beneficial effect was accompanied by affection of the metabolic pathway of TMA-TMAO and reshaping the gut microbiota. Another example of postbiotics use for hypertension of developmental origins is conjugated linoleic acid. One study showed the benefit of conjugated linoleic acid, a gut microbial metabolite derived from dietary polyunsaturated fatty acids, on high-fat-diet-induced hypertension [136].

Moreover, dietary nutrients have also been applied as gut microbiota-targeted therapies for hypertension of developmental origins. Former reviews have sufficiently illustrated the impact of diet on the gut microbiome [142,143]. Prior research demonstrating that specific nutrient intake can be beneficial to protecting from hypertension of developmental origins in various animal models [26,144]. These nutrients include folic acid [145], vitamin E [146], polyunsaturated fatty acids [147], and certain amino acids [27,148,149]. However, very few of them have been studied about their impact in gut microbiota related to developmental hypertension. One recent study showed that L-cysteine therapy protects adult offspring against maternal CKD-induced hypertension, associated with enhancement of beneficial genera *Oscillibacter* and *Butyricicoccus*, as well as depletion of indole-producing genera *Alistipes* and *Akkermansia* [137]. Another study reported that tryptophan supplementation during gestation prevents maternal CKD-induced offspring hypertension. The protective effect of tryptophan supplementation is related to alterations to several tryptophan-metabolizing microbes and the AHR signaling pathway [134].

Notably, increasing evidence supports the notion that altered gut microbial composition and function is evident in hypertension that can be evoked with diets high in salt [83,150,151,152]. Although maternal high salt consumption has been associated with offspring hypertension [29], much remains to be elucidated about the interplay between gut microbiota with high-sodium diets and the role of low-salt diet as a reprogramming strategy for hypertension of developmental origins.

Together, current evidence from animal models supports that modulation of gut microbiota compositions and its derived metabolites through gut microbiota-targeted therapies, in the long term, may enable the capacity to prevent the development of hypertension in a desired favorable direction.

## 6. Conclusions and Perspectives

Animal models have made significant contributions to research in hypertension of developmental origins, giving rise to substantial evidence of an interconnection between early-life insult stimuli, gut microbiota dysbiosis, and hypertension in adulthood. Our review provides insights into the importance of animal models not only in investigating underlying mechanisms behind hypertension of developmental origins, but also in developing early-life gut microbiota-targeted therapy to help prevent hypertension in later life. Considering that hypertension is a major hallmark of metabolic syndrome, aforementioned animal models may be potential models to evaluate developmental programming of metabolic syndrome-related disorders, not to mention that the link between gut microbiota and metabolic syndrome have been extensively discussed in many research papers [153,154].

Animal models are generally considered an intermediate step between bench and human trials. While animal studies are a regulatory requirement for validating preliminary experimental data, animals will remain indispensable in research for some time [155]. However, alternative approaches to animal models need to be investigated and adopted. The integration of computer models with modeling in vitro tissues and organs should be considered as alternative protocols to reduce the use of animals in scientific research [156].

To move the field forward, some unsolved aspects toward clinical translation need to be considered. Despite abovementioned early-life insults having been identified in animal models of developmental hypertension, there may be more risk factors in nature that can adversely influence the BP of adult progeny awaiting to be discovered.

Another important aspect is that the implication of the gut microbiota transfer early in life to the development of hypertension is still unknown, although FMT has been extensively studied in microbiome-associated pathologies, including hypertension [78,81,82]. However, currently, little information exists about their potential application in hypertension of developmental origins.

Animal studies suggest that early use of certain prebiotics, probiotics, or postbiotics may prevent the developmental programming of hypertension, while the exact mechanisms have not been entirely elucidated. What is absent in the literature is whether other prebiotic-rich foods or prebiotic-like components, either individually or in combination, in pregnancy and lactation can also change the gut microbiota to protect adult progeny against hypertension in various animal models.

In conclusion, gut microbiota dysbiosis is a meaningfully pathogenetic link for hypertension of developmental origins. Each of the aforementioned animal models was applied to examine a specific hypothesis, and neither can be considered superior in respect of all aspects of research on hypertension of developmental origins. After all this greater understanding of animal models used for DOHaD research and remarkable growth in gut microbiota-targeted therapies, we believe that translating this growing body of evidence into clinical practice is a valuable strategy to reduce the global hypertension-related burden.

## Figures and Tables

**Table 1:** Animal models reporting hypertension of developmental origins associated with dysbiotic gut microbiota.

Animal Models	Species/Gender	Age at Measure	Alterations of Gut Microbiota	Ref.
Maternal high-fructose diet	SD rat/M	12 weeks	Decreased renal GPR41 and GPR43 expression	[93]
Maternal high-fructose diet	SD rat/M	12 weeks	Decreased plasma TMA level; reduced abundance of genus *Akkermansia* and phylum *Verrucomicrobia*	[94]
Maternal plus post-weaning high-fructose diet	SD rat/M	12 weeks	Decreased abundance of genera *Bacteroides, Dysgonomonas*, and *Turicibacter*	[95]
Maternal high-fructose diet and TCDD exposure	SD rat/M	12 weeks	Increased abundance of genus *Gordonibacter*	[96]
Maternal high-fat and high-cholesterol diet	Wistar rat/M	90 days	Decreased α-diversity	[97]
Maternal plus post-weaning high-fat diet	SD rat/M	16 weeks	An increased F/B ratio; a reduction of genera *Lactobacillus* and *Akkermansia*	[98,99]
Maternal hypertension	SHR/M	12 weeks	An increased abundance of the genera *Bifidobacterium, Lactobacillus, Turicibacter*, and *Akkermansia*	[40]
Maternal hypertension	SHR/M	12 weeks	An increased F/B ratio	[100]
Maternal CKD	SD rat/M	12 weeks	An increased F/B ratio; a reduction of genera *Bifidobacterium, Ruminococcus, Alistipes*; decreased acetate and butyrate in the plasma; and increased plasma TMAO level.	[43]
Maternal dyslipidemia	Wistar rat/M and F	24 weeks	A decrease of genera *Lactobacillus* abundance	[101]
Maternal L-NAME administration plus post-weaning high-fat diet	SD rat/M	16 weeks	An increased F/B ratio	[102]
Maternal minocycline administration	SD rat/M	12 weeks	An increase F/B ratio, and decreased genera *Lactobacillus*, *Ruminococcus*, and *Odoribacter* abundance	[52]
Maternal TMAO and ADMA exposure	SD rat/M	12 weeks	Decreased abundance of *Erysipelotrichaceae* family	[103]
Maternal TCDD exposure	SD rat/M	12 weeks	Decreased α-diversity, and increased F/B ratio, and a decreased abundance of genera *Ruminococcus, Roseburia*, and *Odoribacter*	[104,105]
Prenatal androgen exposure	Wistar rat/F	4 months	An increased abundance of bacteria associated with production of SCFAs.	[106]

Studies tabulated according to animal models, and age at measure; SD = Sprague-Dawley; SHR = spontaneously hypertensive rat; M = male; F = female; CKD = chronic kidney disease; TCDD = 2,3,7,8-tetrachlorodibenzo-p-dioxin; ADMA = asymmetric dimethylarginine; GPR41 = G protein-coupled receptor 41; GPR43 = G protein-coupled receptor 43; TNA = trimethylamine; TMAO = trimethylamine N-oxide; L-NAME = N^G^-nitro-L-arginine-methyl ester; F/B ratio = *Firmicutes* to *Bacteroidetes* (F/B) ratio; SCFA = short chain fatty acid.

**Table 2:** Summary of animal models documenting gut microbiota-targeted therapies for hypertension of developmental origins.

Gut Microbiota-Targeted Therapies	Animal Models	Species/Gender	Age at Evaluation	Ref.
Probiotics				
Daily oral gavage of *Lactobacillus casei* (2 × 10⁸ CFU/day)	Maternal high-fructose diet	SD rat/M	12 weeks	[93]
Daily oral gavage of *Lactobacillus casei* (2 × 10⁸ CFU/day)	Perinatal high-fat diet	SD rat/M	16 weeks	[99]
Prebiotics				
5% *w/w* long chain inulin	Maternal high-fructose diet	SD rat/M	12 weeks	[93]
5% *w/w* long chain inulin	Perinatal high-fat diet	SD rat/M	16 weeks	[99]
Resveratrol (50 mg/L) in drinking water	Maternal TMAO and ADMA exposure	SD rat/M	12 weeks	[103]
Resveratrol (50 mg/L) in drinking water	Perinatal TCDD exposure model	SD rat/M	12 weeks	[105]
Resveratrol (50 mg/L) in drinking water	Maternal adenine-induced CKD	SD rat/M	12 weeks	[113]
Daily oral gavage of garlic oil (100 mg/kg/day)	Perinatal high-fat diet	SD rat/M	16 weeks	[110]
Postbiotics				
Magnesium acetate (200 mmol/L) in drinking water	Maternal high-fructose diet	SD rat/M	12 weeks	[94]
1% DMB in drinking water	Maternal high-fructose diet	SD rat/M	12 weeks	[94]
1% DMB in drinking water	Maternal high-fructose diet and TCDD exposure	SD rat/M	12 weeks	[96]
1% conjugated linoleic acid	Maternal high-fat diet	SD rat/M	18 weeks	[136]
Dietary Nutrients				
Daily oral gavage of tryptophan (200 mg/kg/day)	Maternal adenine-induced CKD	SD rat/M	12 weeks	[134]
Daily oral gavage of L- or D-cysteine (8 mmol/kg/day)	Maternal adenine-induced CKD	SD rat/M	12 weeks	[137]

Studies tabulated based on types of intervention and animal models. TCDD = 2,3,7,8-tetrachlorodibenzo-p-dioxin; CKD = chronic kidney disease; TMAO = trimethylamine-N-oxide; ADMA = asymmetric dimethylarginine; SD = Sprague-Dawley rat; DMB = 3,3-maternal dimethyl-1-butanol.

## Data Availability

Data are contained within the article.
